# Physical Fitness Outcomes in Youth with Autism Spectrum Disorder Following Judo Training in Inclusive Settings with Neurotypical Peers

**DOI:** 10.3390/pediatric18050138

**Published:** 2026-10-12

**Authors:** Pola Jalowska, Adam Prokopczyk, Marek Sokołowski

**Affiliations:** Department of Sport and Defence Education, Poznan University of Physical Education, 61-871 Poznań, Poland; prokopczyk@awf.poznan.pl (A.P.); sokolowski@awf.poznan.pl (M.S.)

**Keywords:** ASD, autism spectrum disorder, martial arts, adapted physical activity, judo, inclusive sport, physical fitness

## Abstract

Background: Physical fitness plays an important role in healthy development; however, children and adolescents with autism spectrum disorder (ASD) typically demonstrate lower physical fitness levels than their neurotypical peers. This study examined changes in physical fitness during 10 months of judo training in inclusive groups. Methods: Ninety children and adolescents aged 7–14 years (36 with ASD and 54 neurotypical peers) participated. Physical fitness was assessed with the EUROFIT test battery at baseline and after 5 and 10 months of training. Results: Both groups significantly improved in overall physical fitness and in most individual tests. The Group × Time interaction was significant only for balance, flexibility, and the total EUROFIT (European Physical Fitness Test Battery) score; in the remaining tests, both groups changed at similar rates. Conclusions: Judo training conducted in inclusive groups may be a valuable form of physical activity for children and adolescents with ASD, as it was accompanied by improvements in physical fitness. The findings also support the feasibility of implementing judo programs in inclusive settings.

## 1. Introduction

Autism spectrum disorder (ASD) is a neurodevelopmental disorder affecting social, communication, and behavioral skills. In addition to difficulties in social communication and interaction, ASD is characterized by restricted and repetitive patterns of behavior, activities, and interests. These behaviors often occur from childhood, hindering daily functioning and usually affecting the normal development of motor skills [1,2]. Contemporary therapeutic measures for people with ASD include non-pharmacological therapies involving behavioral and sensory training [3], therapies based on sports training, as well as pharmacological therapies aimed at treating comorbid disorders, in which psychotropic drugs are used [4]. Recent evidence indicates that exercise-based interventions may improve several aspects of functioning in children and adolescents with ASD, including motor performance and executive functioning [5,6]. Studies have shown that children with autism spectrum disorder have motor problems such as abnormal gait, abnormal muscle tone, balance problems, and poor motor control [7]. Children with ASD are less physically active, exercise less, and have a lower heart rate compared to typically developing children [8,9]. Research indicates that children and adolescents with ASD are less likely to participate in sports activities due to difficulties in social functioning, lack of adequate infrastructure, or lack of preparation on the part of coaches/teachers to work with such individuals [10]. The environment and previous experiences related to sports activities also influence the decision to engage in physical activity [11].

Contemporary scientific research focuses on finding forms of physical activity that have positive effects on people with ASD. It has been shown that children with ASD should start sports training immediately after diagnosis and should participate in physical activities with their neurotypical peers [12], ensuring the proper functioning and development of the cardiovascular and metabolic systems and cardiovascular and respiratory efficiency [13]. Research has suggested that grappling-based activities may offer opportunities for structured physical interaction and engagement among individuals with ASD [14]. It has also been shown that 13 weeks of mixed martial arts (MMA) training significantly improved executive functions in children on the autism spectrum disorder compared to children who did not participate in the classes [15].

Inclusive physical activity refers to participation of individuals with and without disabilities within the same organized physical activity environment, with activities and training conditions adapted, when necessary, to accommodate differences in individual abilities and needs. In the context of children and adolescents with ASD, inclusive sport participation may provide an opportunity to engage in structured physical activity alongside neurotypical peers rather than in separate intervention settings. Research on inclusion in physical education indicates that successful inclusion is influenced by multiple individual, social, and environmental factors, and that the perspectives of participants and other stakeholders are important for understanding the inclusion process [16]. In addition, a systematic review and meta-analysis of children and adolescents with and without ASD in inclusive schools showed that participants with ASD had lower levels of physical activity than their typically developing peers and that individual, social, and environmental factors influenced physical activity participation [17]. Such an approach may therefore be relevant in organized sports, where common activities, rules, and training tasks can be adapted to accommodate participants with different levels of motor and physical ability while maintaining a shared training environment. However, the presence of an inclusive setting alone does not demonstrate social inclusion or improved social functioning, which require specific assessment. In the present study, inclusivity refers specifically to the organization of training in mixed groups comprising children and adolescents with ASD and neurotypical peers who participated in the same judo program.

Previous studies suggest that structured physical activity and martial arts may support motor and executive functioning in children and adolescents with ASD [18,19,20]. The importance of early engagement in physical activity for motor development has also been emphasized [21]. However, the available evidence varies in terms of intervention type, study duration, participant characteristics, and assessed outcomes, which limits direct comparisons between studies. Evidence on martial arts in ASD is growing, but it remains fragmentary. A recent systematic review indicates that martial arts and aquatic training show the most consistent benefits for balance and gross motor skills in children with ASD, although the certainty of the evidence is limited [22], and a systematic review of judo programs for neurodevelopmental disorders suggests physical, social, emotional and cognitive benefits [23]. However, judo studies in ASD have largely focused on behavioral, psychosocial or participation outcomes [24,25], and the study of health-related physical fitness that we are aware of used a long-term adapted judo program delivered exclusively to participants with ASD [13]. Structured martial arts games have also been reported to improve behavioral and motor outcomes in children with ASD [26]. In parallel, inclusive physical activity programs in which children with and without ASD exercised together have been shown to improve the motor and social skills of students with ASD and the attitudes of their peers [27], but these were school-based games programs rather than a martial art. Given that children with ASD typically show lower physical fitness than their neurotypical peers [28,29], it is of practical importance for the planning of inclusive sport to know whether a shared training program is followed by similar or different changes in the two groups. To our knowledge, no previous study has combined a long-term judo program delivered in mixed groups of children with and without ASD, a standardized physical fitness assessment battery, and a formal analysis of between group differences in change over time. The present study was designed to address this gap.

The aim of the study was to analyze changes in the level of physical fitness and its components before, during, and at the end of a 10-month judo training program involving children and adolescents with ASD in an inclusive group with neurotypical peers. All participants included in the final sample participated in the same 10-month judo program. Thus, the study did not involve randomization to an intervention and control group. Randomization was used only to allocate participants to training groups within the predefined age strata.

The authors put forward two research hypotheses:

**H1.**  *Judo training will be associated with a significant increase in physical fitness in individuals with autism spectrum disorder and neurotypical individuals between the beginning and end of the training process.*

**H2.**  *Judo training will be associated with a significant increase in most components of physical fitness in individuals with autism spectrum disorder and neurotypical individuals.*

## 2. Materials and Methods

### 2.1. Participants

The study included 90 individuals (28 girls and 62 boys) aged 7 to 14, including 36 individuals (9.16 ± 1.87) diagnosed with autism spectrum disorder and 54 neurotypical children (8.79 ± 1.82). The demographic and anthropometric characteristics of the participants are presented in Table 1.

Only individuals with normal intellectual functioning and no contraindications to physical activity participated in the study. Only individuals who had not previously practiced any sports and had not attended any additional organized physical activities were eligible for the study.

**Figure 1:**
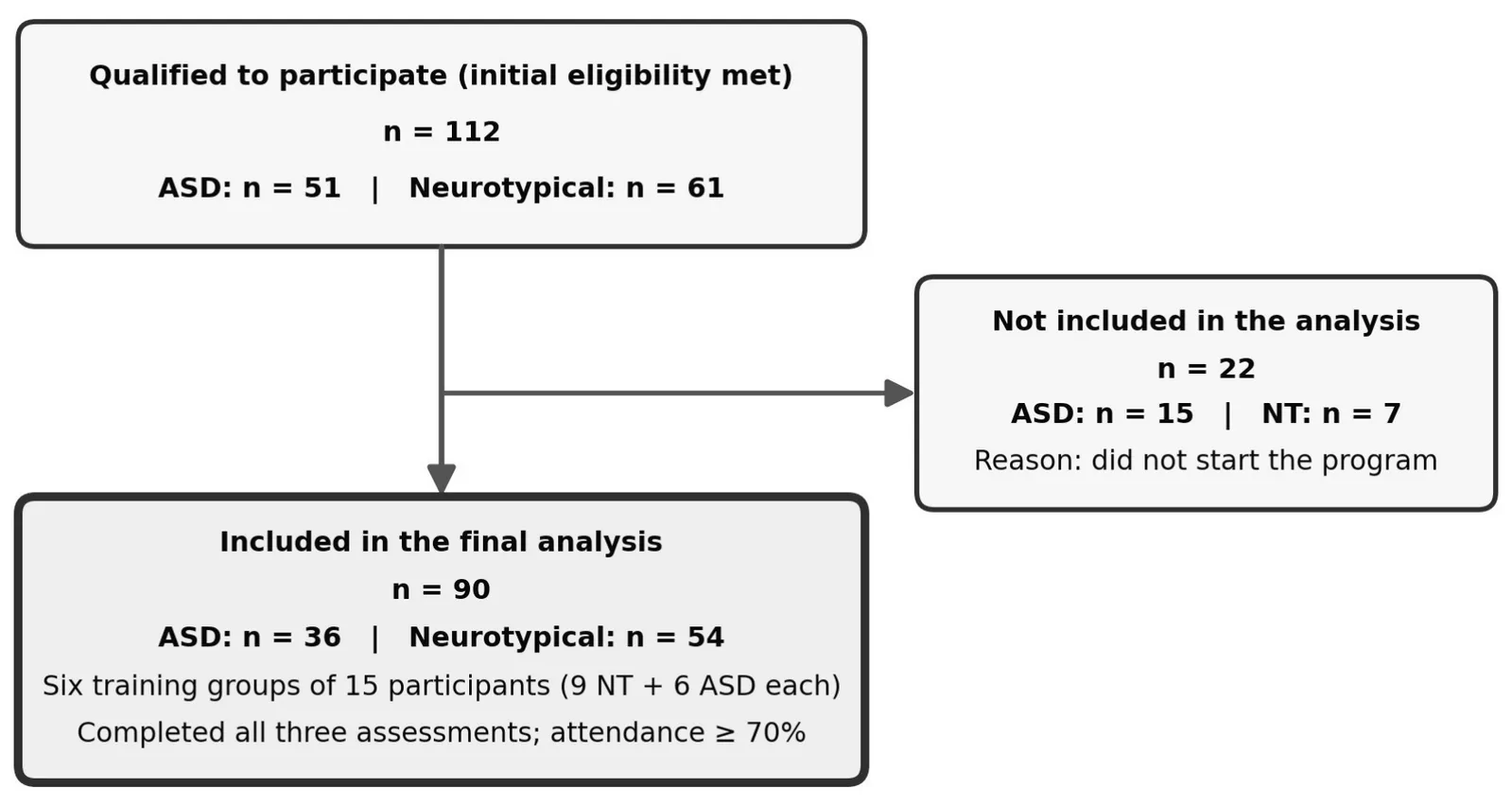
Participant flow and the criteria for inclusion in the final analysis.

A total of 112 individuals were qualified to participate in the study; however, only those who completed all testing sessions and met the predefined minimum attendance requirement of 70% of the judo training sessions were included in the final study sample and statistical analysis. Participants who withdrew from the project and/or whose attendance was below 70% were not included in the final study sample. Of the 112 individuals who met the initial eligibility criteria (51 with ASD and 61 neurotypical), 22 (15 with ASD and 7 neurotypical) did not start the program and were therefore not included in the analysis. The final analyzed sample comprised 90 participants (36 with ASD and 54 neurotypical), who formed six training groups of 15 participants each (9 neurotypical and 6 with ASD per group). The participant flow and the criteria for inclusion in the final analysis are presented in Figure 1.

Participants were divided into training groups, with each group comprising approximately 60% neurotypical participants and 40% participants with ASD. To ensure a comparable age distribution across the training groups, participants were assigned using stratified randomization based on chronological age. Random allocation was performed within two predefined age strata: 7–10 years and 10–14 years. The randomization concerned allocation to training groups only and did not determine ASD status or neurotypical status. Six training groups were established for the 10-month intervention. Each group was designed to comprise 15 participants, including 9 neurotypical participants and 6 participants with ASD. The same two coaches conducted all training sessions throughout the entire intervention period. The composition of the training groups remained unchanged throughout the 10-month training period.

### 2.2. Judo Training Program

The subjects were enrolled in a 10-month judo training program. Judo training sessions were held twice a week in a room equipped for judo classes, covered with a special mat, and lasted 60 min. Each training session consisted of a 12–15 min general warm-up, a 35–40 min main part, and a 5–8 min cool-down. During the training program, the participants took part in general development games and activities, learned the rules and philosophy of judo, how to move on the mat, how to bow, how to fall safely, basic partner techniques (holds and ways to escape them), and standing techniques—throws (*ashi–waza*–foot techniques). The training sessions began and ended with a judo martial arts ceremony.

The program was divided into two parts. The first part lasted for the first five months, and the second part lasted from the sixth to the tenth month. During the first period, the participants learned the rules and philosophy and became accustomed to physical contact with another person. This part was dominated by games and activities involving crawling, wrestling, and moving around the mat in various positions, rolling oneself and one’s training partner, and rolling over another person. During this period, safe falling techniques and basic holds were also taught, along with ways to escape from them. During the second period, the subjects were introduced to their first throws. They perfected the skills they had learned so far, the techniques they had learned on the ground, and learned new ones in a standing position, with the need to use safe falling skills. This period also saw the introduction of simplified training fights.

During training in both periods, balance, jumping, strength, flexibility, and agility exercises were conducted continuously, aimed at increasing the overall physical fitness level of the participants. These exercises were conducted both in the form of games and in a structured manner. The subjects participating in the project received judo uniforms-judogi-as their personal property. During each training session, two coaches were present-the main coach and the assistant coach.

During the study period, all participants also attended regular curricular physical education classes at their respective schools. The content, frequency, intensity, and volume of these classes were not standardized or controlled by the research team.

### 2.3. The European Physical Fitness Test—EUROFIT

The European Physical Fitness Test (EUROFIT) was employed to determine the level of physical fitness. The EUROFIT test was used to measure the level of balance (Flamingo Balance Test), hand movement speed (Plate Tapping Test), explosive power (Standing Broad Jump), flexibility (Sit and Reach Test), static strength (Hand Grip Test), trunk strength (Sit-Ups Test), functional strength (Bent Arm Hang) and agility (10 × 5 m Shuttle Run). The EUROFIT test battery was used to measure physical fitness because it includes scoring tables that assign points based on the performance achieved in a specific trial. The scoring tables are adjusted for both sex and age, with precision down to the quarter of birth.

### 2.4. Flamingo Balance Test

The balance test was performed using a wooden beam measuring 50 × 4 × 3 cm, equipped with two supports (15 × 2 cm each) and end stops to provide stability. Participants were instructed to maintain balance while standing on the beam on one self-selected leg, aligned with the longitudinal axis of the beam. The free leg was flexed at the knee, and the participant had to grasp the foot of the free leg with the hand on the same side, maintaining an upright posture. Before the main trial, participants were allowed to use the shoulder of an assistant to achieve the correct starting position. The trial commenced at the moment the participant released the assistant’s shoulder. The task was to maintain the prescribed position for 60 s. The trial was terminated whenever balance was lost, after which the participant repeated the attempt. The test was evaluated based on the number of attempts required to maintain balance for the full 60 s duration.

### 2.5. Hand Movement Speed Test (Plate Tapping Test)

The hand movement speed test was conducted using a height-adjustable table (or a gymnastic box of equivalent height). Two rubber discs (20 cm in diameter) were horizontally affixed to the table surface, with the centers of the discs positioned 80 cm apart. A rectangular plate (10 × 20 cm) was placed midway between the discs to serve as the central reference area. A digital stopwatch was used for timing. Participants stood in a small straddle stance in front of the table. The non-dominant hand was placed flat on the central rectangular plate, while the dominant hand was positioned on one of the outer discs. Upon the command “ready—start,” participants were instructed to move the dominant hand alternately between the two discs, passing it over the stationary non-dominant hand as quickly as possible. Each disc had to be touched on every movement. One trial consisted of 25 complete cycles (50 total touches). Participants stopped the movement on the command “stop.” To maintain consistency, participants were instructed to count each completed cycle aloud. Each participant performed two trials, with a brief rest interval between them. The best (shortest) time obtained across the two attempts was recorded as the final score.

### 2.6. Explosive Power Test (Standing Long Jump)

The assessment of lower-limb explosive power was performed using two connected gymnastic mats (or an exercise mat), chalk, and a measuring tape. Participants stood in a parallel stance with feet shoulder-width apart, positioned just behind a designated starting line. From this position, they executed a preparatory movement involving a slight forward lean of the trunk, knee flexion, and a backward arm swing. This was immediately followed by a vigorous forward arm swing and a simultaneous two-foot take-off, jumping forward as far as possible. The landing distance was measured from the starting line to the nearest heel mark left on the mat. Each participant performed three jumps, and the longest distance (in centimeters) was recorded as the final result.

### 2.7. Flexibility Test (Sit and Reach Test)

The flexibility test was carried out using a custom-built measuring table (length: 35 cm; height: 32 cm; width: 45 cm) with an extended top surface measuring 55 cm in length. The tabletop extended 15 cm beyond the front vertical board, which served as a foot support. A measurement scale (0–50 cm) was marked along the longitudinal axis of the tabletop with the zero point positioned at the level of the contact between the soles of the feet and the vertical foot-support wall. A movable measuring ruler, approximately 30 cm in length, was placed perpendicularly across the table surface, allowing participants to slide it forward with their hands during trunk flexion. Participants were seated on the floor in an upright position with their legs extended and the soles of the feet placed flat against the front wall of the table (or box). Keeping the knees fully extended, they slowly leaned forward, reaching as far as possible with both hands, and moved the ruler forward along the tabletop in a smooth, continuous motion. Each participant performed two trials. The greater of the two reach distances (measured in centimeters) was recorded as the final result.

### 2.8. Static Strength (Hand Grip Test)

Static muscular strength was assessed using a calibrated hand dynamometer. Participants stood in a small straddle stance with the testing arm extended naturally along the side of the body, ensuring that the hand did not touch the torso during measurement. The dynamometer handle was held firmly in the fingers, adjusted to fit the participant’s hand size. Upon the examiner’s signal, participants performed a brief maximal contraction by squeezing the dynamometer with maximal effort for approximately 2–3 s. The non-testing arm remained relaxed alongside the body. Due to the specific nature of the study group, measurements were taken on both hands. Each hand was tested twice, and that higher result was then selected as the final score, expressed in kilograms (kg) with an accuracy of 1 kg.

### 2.9. Trunk Strength Test (Sit-Ups Test)

Trunk muscle strength was evaluated using the 30 s sit-ups test. Participants lay in a supine position on a gymnastics mat with the knees flexed at a 90° angle and the feet positioned approximately 30 cm apart. The fingers were interlaced and placed behind the head. An assistant kneeling between the participant’s feet stabilized them by pressing gently against the mat. At the examiner’s signal, participants performed repeated sit-ups by lifting the upper body until both elbows touched the knees, then returning immediately to the supine position so that the interlaced fingers briefly contacted the mat. During the movement, participants were instructed not to use their elbows or arms to push off the mat.

Performance was evaluated as the total number of correctly executed sit-ups completed within 30 s.

### 2.10. Functional Strength Test (Bent Arm Hang)

Functional upper-body strength was assessed using the bent arm hang test. Participants stood on a raised platform and grasped a horizontal bar with a pronated (overhand) grip, ensuring that the elbows were bent and the chin positioned above the bar without making contact. Upon removal of the support from under the feet, the participant maintained the initial position for as long as possible without any part of the body touching the bar or swinging excessively. Performance was recorded as the total duration of the hang, measured in seconds, from the moment the support was removed until the chin dropped below the level of the bar.

### 2.11. Agility Test (10 × 5 M Shuttle Run)

Running-Agility were assessed using a 10 × 5 m shuttle run test. Two parallel lines were marked 5 m apart on a flat, non-slip surface. Participants began from a standing split stance position behind the starting line. Upon the command “start,” they sprinted to the opposite line, crossed it completely with both feet, and immediately returned to the starting line. This sequence was repeated until completing a total of ten 5 m segments (five full cycles). Each participant performed the test once. The total time required to complete the 50 m distance (five cycles) was recorded to the nearest 0.1 s using a stopwatch [30,31].

### 2.12. EUROFIT Total Score

The EUROFIT total score was calculated as the sum of the age- and sex- standardized scores obtained for the eight physical fitness tests included in the present study. The results obtained by the participants at each assessment term were converted into age- and sex- adjusted points in accordance with the scoring tables developed for Polish children and adolescents [32,33]. These tables are norm-referenced for Polish school-age children. The scores therefore express the performance of each child relative to Polish neurotypical adolescent population, not raw performance, and the scores of participants with ASD are interpreted against norms for neurotypical children. This should be taken into account when the results are transferred to other countries or time periods (secular trends in physical fitness, differences in anthropometric characteristics and in physical education systems). The Maximal Multistage 20 m Shuttle Run Test was not administered in the presented study because it requires repeated audible signals indicating the start of successive running stage.

The research team considered that these auditory stimuli could cause discomfort or difficulties in following the test protocol among participants with ASD and could therefore interfere with the standardized assessment of aerobic endurance.

### 2.13. Study Protocol

The study protocol involved three measurement periods. Initial data (Term I) were gathered in the first week of the 2024/2025 school year (2–8 September 2024). Subsequent evaluations (Term II) took place after five months of judo training (3–9 February 2025), with the concluding results (Term III) recorded in the final week of the school year (23–29 June 2025).

### 2.14. Statistical Analysis

For all variables studied, the following descriptive statistics were presented for all terms: mean (M); standard deviation (SD); minimum (Min); maximum (Max). The normality of the distribution was checked using the Shapiro–Wilk test. For baseline comparisons between the ASD and neurotypical groups, independent-samples t-tests were used for normally distributed variables, with Welch’s t-test applied when the assumption of homogeneity of variances was not met, whereas the Mann–Whitney U test was used for non-normally distributed variables. The analysis of changes across the three study dates was performed separately for each group using one-way repeated measures ANOVA for parametric variables and Friedman’s ANOVA for non-parametric variables. The effect size for the ANOVA models was examined using eta-squared (η^2^). For variables exhibiting significant change over time in global analysis, pairwise post hoc comparisons between individual test dates were performed using paired-samples t-test post hoc method for parametric data and Wilcoxon’s signed-rank test when the tested variable did not have a normal data distribution in at least one of the dates. No post hoc analysis was performed for variables that did not reach statistical significance in the primary analysis. Cohen’s d coefficient was used to estimate the effect size between the study dates, which was calculated based on Lakens’ formula for parametric variables [34]. For non-parametric post hoc tests, the effect size r was calculated and converted to d to standardize the results [35]. The result was considered insignificant for a test value of less than 0.2, small for a value between 0.2 and 0.49, medium for a value between 0.50 and 0.80, and large for a value above 0.80 [34]. To additionally assess whether the pattern of changes over time differed between the ASD and neurotypical peers, linear mixed-effects models were fitted separately for each physical fitness variable using maximum likelihood (SPSS Statistics, version 28). Group, Time (Term I, Term II, Term III) and the Group × Time interaction were included as categorical fixed effects, while participant was included as a random intercept to account for the repeated measurements within individuals. Multiplicity was controlled using the Bonferroni correction across the nine outcomes (eight fitness tests and the EUROFIT total; α = 0.05/9 = 0.0056), applied to the baseline comparisons, the omnibus analyses (in each group separately) and the Group × Time interactions in the mixed-effects models. Post hoc comparisons for variables meeting this criterion were evaluated at *p* < 0.0167 (0.05/3 pairs of terms). Fixed effects are reported as unstandardized coefficients (β, in EUROFIT points) with standard errors and Wald 95% confidence intervals, which are not adjusted for multiplicity. The overall Group × Time effect was evaluated with a Wald χ^2^ test (2 df), and its magnitude is expressed as approximate partial eta-squared (η^2^p) derived from the Wald statistic, with 95% confidence intervals based on the noncentral F distribution values of ≈0.01, ≈0.06 and ≈0.14 were interpreted as small, medium and large, respectively. Statistica version 13.3 software was used to perform the statistical analysis (StatSoft, Inc., 2015, Tulsa, OK, USA).

## 3. Results

Children and adolescents with ASD achieved higher overall EUROFIT scores on subsequent test dates, showing improvement or maintaining their performance (the latter only in the Hand Grip Test). Neurotypical peers scored higher on the EUROFIT test and improved across most trials at each consecutive assessment. However, over the course of the training program, the difference between the groups in the mean EUROFIT total score decreased from 54.55 pts at Term I to 43.33 pts at Term II and 38.95 pts at Term III. Moreover, this pattern should not be interpreted as a general narrowing of the gap across all physical fitness components, because the Group × Time interaction was statistically significant only for the Flamingo Balance Test, the Sit and Reach Test, and the EUROFIT total score (see Section 3.5). Furthermore, in the Sit and Reach Test, subjects with ASD outperformed their neurotypical peers during Terms II and III (Table 2).

### 3.1. Baseline Analysis

At baseline (Term I), the neurotypical group had higher scores than the ASD group in the Flamingo Balance Test, Standing Broad Jump, Bent Arm Hang, Shuttle Run 10 × 5 m, and EUROFIT total score. No statistically significant baseline differences after correction were observed for the Plate Tapping Test, Sit and Reach Test, Hand Grip Test, or Sit-Ups Test (Table 3).

### 3.2. General Analysis

Both the ASD and neurotypical groups showed significant improvements in overall physical fitness across the study period. Significant improvements were also observed in most individual physical fitness tests, although the pattern of change varied between groups and tests. Between Term 1 and Term 3, significant improvements were observed for the EUROFIT total score and for all individual tests that reached statistical significance in the primary within-group analysis.

### 3.3. ASD Group Analysis

Between Term 1 & Term 2, the ASD group showed significant improvements in overall physical fitness and in most tests. No significant changes were observed in Plate Tapping Test (*p* = 0.1992), Standing Broad Jump (*p* = 0.0398) and Shuttle Run 10 × 5 m (*p* = 0.0238). Between Term 2 & Term 3 significant improvements were observed in overall physical fitness and in all tests, including a moderate increase in the Shuttle Run 10 × 5 m (*p* = 0.0141).

It is worth noting that in the Plate Tapping Test within the ASD group the mean score increased by 1.75 pts between Term I and Term II (39.72 to 41.47) but by a further 4.09 pts between Term II and Term III (41.47 to 45.56). Both comparisons involving Term III were significant (Term I–Term III, *p* = 0.0001; Term II–Term III: *p* = 0.0005). The global effect therefore reflects mainly the improvement in the second half of the program, whereas the change during the first five months did not reach the adjusted significance threshold.

### 3.4. Non-ASD Group Analysis

Between Term 1 & Term 2, the non-ASD group showed significant improvements in overall physical fitness and in most of the assessed tests. Significant improvements were observed in the Flamingo Balance Test (*p* = 0.0010), Sit and Reach Test (*p* = 0.0019), Sit-Ups Test (*p* < 0.0001), Bent Arm Hang (*p* = 0.0005), Shuttle Run 10 × 5 m (*p* = 0.0040), and EUROFIT total score (*p* < 0.0001). No significant changes were observed in the Plate Tapping Test (*p* = 0.0326) and Standing Broad Jump (*p* = 0.1414). Between Term 2 & Term 3, overall physical fitness and all tests showed significant improvement, with the exception of the Shuttle Run 10 × 5 m, which showed no significant change (*p* = 0.1606), (Table 4).

The Hand Grip Test was not included in the pairwise post hoc analysis because the primary global test did not reach the Bonferroni-adjusted significance threshold (Table 2).

### 3.5. Between-Group Analysis of Changes over Time

The linear mixed-effects models showed that the pattern of change across the three assessment dates differed significantly between the ASD and non-ASD groups for the Flamingo Balance Test, the Sit and Reach Test, and the overall EUROFIT score. he models included Group, Time, and the Group × Time interaction as fixed effects. A significant Group × Time interaction was observed for the Flamingo Balance Test (χ^2^ (2) = 75.62, *p* < 0.001), the Sit and Reach Test (χ^2^ (2) = 14.19, *p* = 0.0008), and the EUROFIT total score (χ^2^ (2) = 12.44, *p* = 0.0020). All three interactions remained statistically significant after Bonferroni correction for the nine analyzed variables. No significant Group × Time interaction was observed for the Plate Tapping Test (χ^2^ (2) = 0.20, *p* = 0.904), Standing Broad Jump (χ^2^ (2) = 0.29, *p* = 0.864), Hand Grip Test (χ^2^ (2) = 0.19, *p* = 0.910), Sit-Ups Test (χ^2^ (2) = 1.33, *p* = 0.514), Bent Arm Hang (χ^2^ (2) = 0.62, *p* = 0.735), or Shuttle Run 10 × 5 m (χ^2^ (2) = 0.48, *p* = 0.788). Thus, for these variables, the observed changes over time did not differ statistically between the ASD and non-ASD groups. Thus, statistically significant differences in the longitudinal pattern of change between the two groups were observed only for balance, flexibility, and overall EUROFIT performance (Table 5). For the other six variables, the groups changed at statistically similar rates, and these results should not be interpreted as evidence of convergence between the groups. The interaction coefficients with their standard errors and 95% confidence intervals, together with the effect sizes (η^2^p), are given in Table 6.

## 4. Discussion

The study analyzed the impact of a 10-month judo training program on the physical fitness levels of children and adolescents with autism spectrum disorder and their neurotypical peers training together in inclusive groups. The structured training program was associated with significant improvements in physical fitness in both groups. These results confirm the first research hypothesis. Notably, both groups exhibited a significant increase in physical fitness halfway through the program (after 5 months), at its conclusion (after 10 months), and between the mid-term assessment and final assessments.

The results obtained are consistent with previous studies in non-inclusive groups and confirm that exercise interventions with certain repetitive parts and structure are highly effective in enhancing the physical fitness of youth with ASD. Pan et al. [18] showed that a 12-week physical activity program requiring coordinated movement, focus, and an orderly sequence of movements improved both motor skills and executive functions in children and adolescents with ASD. Phung and Goldberg [15] demonstrated that a 13-week mixed martial arts program significantly improved executive functions in children with ASD compared to a control group, highlighting the role of structured, rule-based combat sports in supporting cognitive self-regulation. Pierantozzi et al. [13] similarly reported health-related physical fitness gains following a long-term adapted judo program in youth with ASD; however, that program was delivered exclusively to participants with ASD and did not include a neurotypical comparison group training alongside them, and it did not report a formal statistical test of the between-assessment change comparable to the Group × Time interaction analysis used here. Relative to both Pierantozzi et al. [13] and Phung and Goldberg [15], the present study involved a considerably longer intervention (10 months versus 13 weeks or shorter reported durations), a larger and mixed-diagnosis sample, and, uniquely, an inclusive training format combining ASD and neurotypical participants, which may partly explain the more comprehensive pattern of fitness improvements observed across nearly all EUROFIT components in the present sample. Furthermore, a meta-analysis indicated that for physical exercise to be effective in treating people with ASD, it must last more than four weeks and be performed at least twice a week, with martial arts showing a particularly positive effect [6]. Considering that neurotypical individuals have a higher level of physical fitness than individuals with ASD [9] and the results of the research, participation in the same judo training program in an inclusive group was associated with regular improvements in physical fitness in both groups. At the same time, it should be noted that despite a significant increase in the level of physical fitness at each of the study dates, the difference between the groups gradually decreased, although neurotypical peers still achieved higher scores in every trial. However, it is important to keep in mind that this descriptive decrease does not reflect a general diminishing of between group differences. The Group × Time interaction was significant only for balance, flexibility, and the total score, while in the remaining tests, both groups improved at statistically similar rates.

Our findings demonstrate substantial and robust improvements across all dimensions of physical fitness in both groups, with the sole exception of Static Strength. This partially confirms the second research hypothesis. The results indicate that substantial improvements in physical fitness were observed during the initial phase of the judo training program, with changes occurring across most of its components. Importantly, nearly all tested components showed significant and strong improvements at the 5-month mark and again at 10 months. Moreover, the magnitude of these changes was predominantly characterized as strong across all study periods. These outcomes resonate with prior research indicating that judo serves as a comprehensive activity for the motor development of children and adolescents [36]. Morales et al. [19] further demonstrated that properly conducted judo training, tailored to individual needs, effectively develops motor skills in youth with ASD. The above reports and our findings suggest that the earliest possible engagement in sports activities may exert a significant impact on the comprehensive motor development of children with ASD [21].

A finding that merits particular attention is that, in the Sit and Reach Test, the ASD group obtained higher scores than the neurotypical group at Term II and Term III, in contrast to the general pattern observed for most other physical fitness components. A plausible factor contributing to this result is the elevated prevalence of generalized joint hypermobility among individuals with ASD compared to their neurotypical peers [29]. Greater joint hypermobility could increase trunk-forward flexion distance in this task independently of training-related adaptations, while also interacting with individual differences in limb-to-trunk proportions. However, because joint mobility was not evaluated independently of this test, this explanation cannot be confirmed and remains speculative. Nonetheless, this highlights the need for future studies to incorporate independent measures of flexibility and joint mobility to help clarify the source of this between-group difference.

When contrasted with previous exercise interventions, the results of this study suggest that well-structured, engaging, and formulaic martial arts programs may offer substantial supportive potential for children and adolescents with ASD [20]. Crucially, our study provides a novel insight by demonstrating that introductory judo training was conducted in inclusive groups comprising participants with ASD and neurotypical peers, with improvements in physical fitness observed in both groups. These findings indicate that a common judo training program can be implemented with participants with ASD and neurotypical peers within the same training environment, although the social acceptability, feasibility, and broader social inclusion outcomes of this approach were not formally assessed.

### 4.1. Study Limitation

Despite the significant and promising findings, this study is not without limitations. First, the absence of a control group—either a passive (sedentary) group or an active group participating in a non-martial arts intervention—limits the ability to determine whether the observed changes in physical fitness were specifically attributable to judo training, as opposed to maturation, natural developmental changes, or other factors associated with participation in physical activity. This design choice was necessitated by the primary aim of assessing the inclusive group environment and the limited sample size of participants with ASD. Second, the rigorous inclusion criteria required participants to have no prior sports experience and to attend all testing sessions, which reduced the final sample size and may limit the generalizability of the results to the broader ASD population, potentially introducing a selection bias. Furthermore, the study did not account for certain uncontrolled confounding variables; the participants’ emotional and physical states, such as baseline fatigue, anxiety, and uncertainty during the testing sessions, could have varied and influenced performance. Additionally, despite the initial screening criteria, it was impossible to fully monitor whether some individuals initiated other organized physical activities outside of this project during the 10-month period. Moreover, the generalisability and ecological validity of the findings may be limited by the specific characteristics of the study setting and participant sample. The intervention was conducted within a structured and organised inclusive training environment, with participants intentionally assigned to training groups comprising approximately 60% neurotypical and 40% participants with ASD. In addition, the inclusion criterion requiring no previous sports experience resulted in a relatively specific participant profile. Therefore, the extent to which the observed findings can be generalised to children and adolescents with different levels of previous physical activity or to less structured settings, such as schools, sports clubs, or more heterogeneous inclusive groups, remains uncertain. Another limitation concerns the participants’ regular curricular physical education. All participants attended physical education classes at their respective schools during the study period. However, the participants came from different schools, and these classes were conducted by different teachers and could therefore vary in terms of teaching methods, content, intensity, and volume of physical activity. The researchers had no control over or standardized information on the content and intensity of these classes, and their potential contribution to changes in physical fitness could not be isolated from the effects of the judo training program. In addition, the timing of the assessments across the school year may have coincided with natural developmental changes and variations in physical activity associated with the school calendar, which should be considered when interpreting the longitudinal changes observed in the study. Another limitation is that the study did not include a formal assessment of the social validity or acceptability of the training program. No structured measures of participant or parent satisfaction were collected, and perspectives of parents, coaches, or teachers regarding the organisation and perceived value of the inclusive training were not systematically assessed. In addition, no qualitative data were collected to explore the reasons for participant withdrawal or non-adherence to the training program. Consequently, although the study was conducted in an inclusive training setting, the present findings do not allow conclusions to be drawn regarding the social acceptability, satisfaction, or broader social validity of the program. Finally, the study did not include a follow-up assessment after completion of the 10-month judo training program. Therefore, it remains unknown whether the observed improvements in physical fitness were maintained after the intervention ended, diminished over time, or returned toward baseline levels. Further studies including post-intervention follow-up assessments are needed to determine the long-term sustainability of the observed effects. An additional limitation concerns the considerable individual variability in motor competence, developmental level, and symptom severity that characterizes the ASD population. Motor coordination deficits in ASD have been reported to be pervasive but highly heterogeneous in magnitude across individuals [37]. The present study did not stratify participants with ASD by developmental level, co-occurring motor impairment, or symptom severity, and this heterogeneity could not be accounted for in the analysis. It should therefore be considered when interpreting the motor-test results for the ASD group and when generalizing the findings to the wider ASD population. Finally, the study assessed only physical fitness outcomes and did not include measures of potential indirect effects of judo participation, such as social skills, emotional regulation, self-esteem, or repetitive behaviors, which have been reported in relation to martial-arts-based interventions in previous studies. Additional limitations relate to the physical fitness measurement. The same test battery was administered on three occasions. A practice or familiarization effect therefore cannot be excluded: part of the improvement may reflect greater familiarity with the test procedures, equipment and testing environment, a better understanding of the tasks or lower test-related anxiety, rather than genuine changes in physical fitness. This may be especially relevant for participants with ASD, for whom unfamiliar tasks, settings and examiners may affect performance.

### 4.2. Future Study

Future research should aim to explore the neural and sensorimotor mechanisms underlying functional adaptation in diverse environments, as well as to determine the optimal training frequency and duration, while tailoring teaching methods and formats to optimize outcomes for individuals with ASD. A comparative analysis involving both a sedentary control group and a group participating in non-martial arts activities would also be justified—an approach that was unfeasible in the present study due to the limited sample size of participants with ASD. Given the continuous and significant increase in fitness levels observed here, it would be reasonable to conduct multi-year longitudinal observations. This would allow for an assessment of developmental trajectories and for testing whether, and in which components of physical fitness, the gap between individuals with ASD and their neurotypical peers narrows, given that in the present study differential change was observed only for balance, flexibility and the total score.

## 5. Conclusions

The findings demonstrate that judo training was associated with significant improvements in physical fitness across the study period in both children and adolescents with autism spectrum disorder (ASD) and their neurotypical peers. Specifically, improvements were observed in balance, hand movement speed, explosive power, flexibility, trunk strength, functional strength, and running agility whereas static strength did not show a significant change across the study period. The comparison of change between the groups showed greater gains in the ASD group only for balance, flexibility and the total EUROFIT score, whereas in the remaining tests the two groups changed at similar rates. The data therefore do not support a general convergence of the physical fitness of the two groups. These results suggest that judo training may serve as a valuable form of physical activity for supporting the development of physical fitness in youth with ASD. The present study also demonstrates that such training can be delivered in inclusive groups comprising participants with ASD and neurotypical peers, although the social acceptability, feasibility, and broader social inclusion outcomes of this approach were not formally assessed.

## Figures and Tables

**Table 1:** Demographic and anthropometric characteristics of the study participants with ASD (*n* = 36) and neurotypical participants (*n* = 54).

Variable	ASD M ± SD	Neurotypical M ± SD
Age [years]	9.16 ± 1.87	8.79 ± 1.82
Body weight [kg]	38.12 ± 12.56	34.49 ± 11.20
Height [cm]	139.60 ± 13.63	137.58 ± 13.39

**Table 2:** Descriptive statistics and comparison of physical fitness test results in Term I, Term II and Term III for children and adolescents with ASD (*n* = 36) and neurotypical peers (*n* = 54).

	Term I	Term II	Term III			
Variable	M ± SD (Min–Max)	M ± SD (Min–Max)	M ± SD (Min–Max)	Tests Result	*p*	η^2^
ASD (n = 36)						
Flamingo Balance Test [pts]	39.69 ± 11.91 (17–61)	47.14 ± 12.37 (25–69)	54.14 ± 11.18 (32–69)	64.64 ^b^	**<0.0001**	0.90
Plate Tapping Test [pts]	39.72 ± 7.81 (19–54)	41.47 ± 7.90 (23–58)	45.56 ± 9.80 (28–74)	17.64 ^a^	**<0.0001**	0.34
Standing Broad Jump [pts]	47.97 ± 11.51 (29–74)	50.31 ± 10.49 (30–80)	53.64 ± 10.75 (33–83)	18.48 ^a^	**<0.0001**	0.35
Sit and Reach Test [pts]	42.72 ± 12.05 (17–65)	46.28 ± 11.26 (20–67)	50.36 ± 10.77 (29–71)	58.45 ^a^	**<0.0001**	0.63
Hand Grip Test [pts]	49.61 ± 7.50 (31–63)	49.61 ± 7.61 (30–69)	50.72 ± 7.59 (30–68)	1.48 ^a^	0.2351	0.04
Sit-Ups Test [pts]	37.00 ± 10.16 (11–59)	42.22 ± 11.95 (17–62)	46.61 ± 11.93 (19–66)	52.31 ^a^	**<0.0001**	0.60
Bent Arm Hang [pts]	26.97 ± 20.99 (0–55)	34.19 ± 18.00 (0–56)	38.36 ± 16.74 (0–59)	44.02 ^b^	**<0.0001**	0.61
Shuttle Run 10 × 5 m [pts]	47.08 ± 10.19 (15–66)	49.83 ± 9.89 (21–68)	51.36 ± 9.74 (23–68)	13.81 ^a^	**<0.0001**	0.28
EUROFIT total [pts]	330.78 ± 62.99 (177–449)	361.06 ± 61.15 (210–493)	390.75 ± 57.12 (271–534)	167.03 ^a^	**<0.0001**	0.83
Neurotypical Peers (*n* = 54)						
Flamingo Balance Test [pts]	55.46 ± 8.78 (34–70)	57.39 ± 7.64 (41–69)	60.56 ± 6.27 (44–69)	24.04 ^b^	**<0.0001**	0.22
Plate Tapping Test [pts]	43.31 ± 8.43 (21–66)	45.26 ± 8.45 (28–65)	48.80 ± 8.59 (30–66)	26.54 ^a^	**<0.0001**	0.33
Standing Broad Jump [pts]	54.72 ± 8.99 (39–72)	56.37 ± 9.65 (35–76)	60.17 ± 9.12 (42–82)	20.90 ^a^	**<0.0001**	0.28
Sit and Reach Test [pts]	43.44 ± 11.24 (22–65)	45.44 ± 10.96 (21–66)	47.72 ± 9.89 (25–68)	28.49 ^a^	**<0.0001**	0.35
Hand Grip Test [pts]	50.91 ±7.12 (33–71)	51.02 ± 7.32 (35–70)	52.39 ± 6.79 (37–76)	4.93 ^a^	0.0090	0.09
Sit-Ups Test [pts]	42.91 ± 10.70 (14–72)	46.69 ± 9.90 (21–69)	51.85 ± 10.55 (23–73)	59.61 ^a^	**<0.0001**	0.53
Bent Arm Hang [pts]	38.48 ± 18.64 (0–64)	44.07 ± 13.99 (0–74)	47.87 ± 12.69 (0–77)	46.18 ^b^	**<0.0001**	0.43
Shuttle Run 10 × 5 m [pts]	56.09 ± 6.24 (42–70)	58.15 ± 7.56 (42–73)	60.35 ± 6.11 (49–78)	15.29 ^a^	**<0.0001**	0.22
EUROFIT total [pts]	385.33 ± 46.89 (272–482)	404.39 ± 43.63 (291–484)	429.70 ± 42.27 (304–514)	105.35 ^a^	**<0.0001**	0.67

Note: ^a^—one-way repeated measures ANOVA, ^b^—Friedman’s ANOVA, significant results (*p* < 0.0056) are highlighted in bold.

**Table 3:** Baseline comparison of physical fitness measures between children and adolescents with ASD and neurotypical peers (Term I).

Variable	ASD M ± SD	Neurotypical M ± SD	Results	*p*
Flamingo Balance Test [pts]	39.69 ± 11.91	55.46 ± 8.78	−6.81 ^a^	**<0.0001**
Plate Tapping Test [pts]	39.72 ± 7.81	43.31 ± 8.43	−2.07 ^a^	0.0418
Standing Broad Jump [pts]	47.97 ± 11.51	54.72 ± 8.99	−2.97 ^a^	**0.0043**
Sit and Reach Test [pts]	42.72 ± 12.05	43.44 ± 11.24	−0.29 ^a^	0.7756
Hand Grip Test [pts]	49.61 ± 7.50	50.91 ± 7.12	−0.82 ^a^	0.4150
Sit-Ups Test [pts]	37.00 ± 10.16	42.91 ± 10.70	−2.65 ^a^	0.0099
Bent Arm Hang [pts]	26.97 ± 20.99	38.48 ± 18.64	628.00 ^b^	**0.0043**
Shuttle Run 10 × 5 m [pts]	47.08 ± 10.19	56.09 ± 6.24	−4.74 ^a^	**<0.0001**
EUROFIT total [pts]	330.78 ± 62.99	385.33 ± 46.89	−4.44 ^a^	**<0.0001**

Note: ^a^—Welch’s t-test; ^b^—Mann–Whitney U, significant results (*p* < 0.0056) are highlighted in bold.

**Table 4:** Pairwise post hoc comparisons and effect sizes for changes in physical fitness test results between Term I, Term II and Term III in children and adolescents with ASD (*n* = 36) and neurotypical peers (*n* = 54).

Variable	Term 1 & 2		Term 1 & 3		Term 2 & 3	
	*p*	d	*p*	d	*p*	d
ASD (*n* = 36)						
Flamingo Balance Test [pts] ^b^	**<0.0001**	3.24	**<0.0001**	3.56	**<0.0001**	2.95
Plate Tapping Test [pts] ^a^	0.1992	0.59	**0.0001**	0.95	**0.0005**	1.28
Standing Broad Jump [pts] ^a^	0.0398	0.81	**0.0010**	1.33	**0.0020**	2.24
Sit and Reach Test [pts] ^a^	**0.0001**	2.15	**0.0001**	4.04	**0.0001**	3.06
Hand Grip Test [pts] ^a^	**-**	-	**-**	-	**-**	-
Sit-Ups Test [pts] ^a^	**0.0001**	1.42	**0.0001**	3.75	**0.0002**	2.30
Bent Arm Hang [pts] ^b^	**0.0004**	1.43	**<0.0001**	2.42	**<0.0001**	1.83
Shuttle Run 10 × 5 m [pts] ^a^	0.0238	1.71	**0.0001**	1.26	**0.0141**	0.65
EUROFIT total [pts] ^a^	**0.0001**	4.86	**0.0001**	6.91	**0.0001**	8.07
Neurotypical Peers (*n* = 54)						
Flamingo Balance Test [pts] ^b^	**0.0010**	1.00	**<0.0001**	1.50	**<0.0001**	1.36
Plate Tapping Test [pts] ^a^	0.0326	0.77	**0.0001**	1.15	**0.0001**	0.94
Standing Broad Jump [pts] ^a^	0.1414	0.37	**0.0001**	0.89	**0.0002**	1.55
Sit and Reach Test [pts] ^a^	**0.0019**	1.28	**0.0001**	2.42	**0.0004**	1.75
Hand Grip Test [pts] ^a^	**-**	-	**-**	-	**-**	-
Sit-Ups Test [pts] ^a^	**0.0001**	1.07	**0.0001**	1.91	**0.0001**	2.27
Bent Arm Hang [pts] ^b^	**0.0005**	1.07	**<0.0001**	1.94	**<0.0001**	1.85
Shuttle Run 10 × 5 m [pts] ^a^	**0.0040**	0.32	**0.0001**	0.84	0.1606	0.85
EUROFIT total [pts] ^a^	**0.0001**	1.49	**0.0001**	2.65	**0.0001**	3.49

Note: ^a^—paired-samples t-test post hoc, ^b^—Wilcoxon signed-rank test, significant results (*p* < 0.0167) are highlighted in bold.

**Table 5:** Group × Time interaction effects for physical fitness measures in children and adolescents with ASD (*n* = 36) and neurotypical peers (*n* = 54).

Variable	Group × Time χ^2^ (2)	*p*	Bonferroni
Flamingo Balance Test [pts]	75.62	<0.001	Significant
Plate Tapping Test [pts]	0.20	0.904	Not significant
Standing Broad Jump [pts]	0.29	0.864	Not significant
Sit and Reach Test [pts]	14.19	0.0008	Significant
Hand Grip Test [pts]	0.19	0.910	Not significant
Sit-Ups Test [pts]	1.33	0.514	Not significant
Bent Arm Hang [pts]	0.62	0.735	Not significant
Shuttle Run 10 × 5 m [pts]	0.48	0.788	Not significant
EUROFIT total [pts]	12.44	0.0020	Significant

Note: χ^2^ (2)—Wald test statistic for the Group × Time interaction; *p* < 0.0056 indicates statistical significance after Bonferroni correction.

**Table 6:** Group × Time interaction coefficients and effect sizes from linear mixed-effects models for children and adolescents with ASD (*n* = 36) and neurotypical peers (*n* = 54).

Variable	ASD × Term II β (SE) [95% CI]	ASD × Term III β (SE) [95% CI]	Group × Time Partial η^2^ [95% CI]
Flamingo Balance Test [pts]	+5.52 (1.08) [3.40; 7.64]	+9.35 (1.08) [7.23; 11.47]	0.296 [0.185; 0.390]
Plate Tapping Test [pts]	−0.19 (1.23) [−2.61; 2.22]	+0.35 (1.23) [−2.06; 2.76]	0.001 [0.000; 0.015]
Standing Broad Jump [pts]	+0.69 (1.29) [−1.85; 3.22]	+0.22 (1.29) [−2.31; 2.75]	0.002 [0.000; 0.020]
Sit and Reach Test [pts]	+1.56 (0.89) [−0.19; 3.31]	+3.36 (0.89) [1.61; 5.11]	0.073 [0.013; 0.149]
Hand Grip Test [pts]	−0.11 (0.88) [−1.83; 1.61]	−0.37 (0.88) [−2.09; 1.35]	0.001 [0.000; 0.014]
Sit-Ups Test [pts]	+1.44 (1.25) [−1.01; 3.90]	+0.67 (1.25) [−1.79; 3.12]	0.007 [0.000; 0.042]
Bent Arm Hang [pts]	+1.63 (2.71) [−3.69; 6.95]	+2.00 (2.71) [−3.32; 7.32]	0.003 [0.000; 0.029]
Shuttle Run 10 × 5 m [pts]	+0.69 (1.15) [−1.55; 2.94]	+0.02 (1.15) [−2.23; 2.27]	0.003 [0.000; 0.026]
EUROFIT total [pts]	+11.22 (4.56) [2.28; 20.17]	+15.60 (4.56) [6.66; 24.55]	0.065 [0.009; 0.138]

Note: β—unstandardized coefficient; SE—standard error; 95% CI—Wald confidence interval.

## Data Availability

Data are available upon request to the corresponding author.
